# Hybrid Assembly from 75 E. coli Genomes Isolated from French Bovine Food Products between 1995 and 2016

**DOI:** 10.1128/mra.01095-22

**Published:** 2023-02-01

**Authors:** Sandra Jaudou, Mai-Lan Tran, Fabien Vorimore, Patrick Fach, Sabine Delannoy

**Affiliations:** a Pathogenic E. coli Unit, Laboratory for Food Safety, Anses, Maisons-Alfort, France; b IdentyPath Genomics Platform, Laboratory for Food Safety, Anses, Maisons-Alfort, France; University of Maryland School of Medicine

## Abstract

Here, we report the complete (or near-complete) genome sequences of 75 Escherichia coli isolates, including 71 *stx*-positive E. coli isolates, isolated in France between 1995 and 2016 from food of bovine origin. Genomes were assembled using a combination of long- and short-read sequencing.

## ANNOUNCEMENT

Shiga toxin-producing Escherichia coli (STEC) food contamination can lead to severe human symptoms (1). Here, we assembled 75 E. coli genomes, including 31 *eae*-positive STEC and 39 *eae*-negative STEC genomes, isolated from food of bovine origin within a 21-year period in France.

E. coli strains were originally isolated on Trypticase soy broth with yeast extract (TSYE) plates overnight at 37°C. Isolates were revived from 20 to 30% glycerol stock on TSYE plates and incubated overnight at 37°C. One colony was cultured overnight at 37°C in brain heart infusion broth (BHI) with rotation. Genomic DNA was extracted from BHI culture (1 mL) using different extraction methods and sequenced using both MiSeq (Illumina, Inc., San Diego, CA, USA) and MinION (Nanopore, Oxford Science Park, Oxford, UK) technologies (2). Default parameters were used for all software unless otherwise specified. Illumina libraries were constructed using Nextera XT kit and sequenced on v2 micro or standard flow cells (2 × 150 bp or 2 × 250 bp). MinION libraries were constructed from unsheared and non-size-selected DNA using the SQK-LSK109 ligation kit and EXP-NBD104 or EXP-NBD114 barcoding kit and sequenced on FLO-MIN106 flow cells using an Mk1B device.

Illumina reads were trimmed and quality checked using the CLC Genomics Workbench v21 (Qiagen). We generated 299,212 to 3,972,904 reads (mean, 1,368,471 ± 720,963.42) with an average read length of 198.08 (±37.99) bases per sample, representing 12 to 174× (mean, 51 ± 22.65) (Table 1). MinION FAST5 files were basecalled and demultiplexed using guppy_basecaller and guppy_barcoder v3.4.5+fb1fbfb or v5.0.11+2b6dbff using “*--*compress-fastq” and “--trim-barcodes” parameters (3). Statistics on raw reads were performed using NanoPlot v1.29.0 (4). Between 14,450 and 1,992,878 reads (mean, 239,919 ± 341,570.565) were generated with *N*_50_ values ranging from 2,565 bp to 29,351 bp (mean,16,330 ± 7,056.38). Assemblies were generated using Unicycler v0.4.8 (*--*min_fasta_length 1,000), Flye v2.6 or v2.8.1-b1676 (--genome-size 5m), and Raven v1.2.2 (5–7). Assembly quality and contiguity were assessed using QUAST v5.0.2 (8) and manual inspection of the assemblies (*stx*-phage and large plasmids integrity). Either the Unicycler-generated assembly was conserved or the Flye and/or Raven assemblies were further polished with short reads using racon v1.4.13, medaka v0.12.1 (optional), and pilon v1.24 polishing tools (3 to 5 rounds) (9, 10; https://github.com/nanoporetech/medaka). If none of these strategies produced satisfying assemblies, Canu v1.8 or v2.1.1 (genomeSize 5m) or Unicycler based on Flye or Raven assembly (--existing_long-read_assembly) was generated (11). Because assemblers have different performances regarding plasmid or phage reconstruction, several assemblies were retained for some of the samples. Whenever possible, Unicycler performed the circularization of the replicons (5).

**TABLE 1:** Assembly metrics of Escherichia coli draft genomes and NCBI accession numbers for each isolate sequenced

NCBI accession no. (ONT, Illumina)	DNA extraction method(s)^*j*^	Strain	Serotype^*k*^	*stx* subtype(s)	*eae*	Hybrid assembly	MinION read length (*N*_50_ [bp])	No. of contigs	Genome size (bp)	Assembly *N*_50_ (bp)	GC content (%)	Genome coverage (×)	GenBank accession no.
SRR18191646, SRR18191606	Bead based	1429	O76:H19	1c	−	Unicycler	10,334	3	5,316,065	5,183,674	50.64	107	JAOQMI000000000
						Canu^*a*^		5	5,528,843	5,270,995	50.64	107	JAOWPQ000000000
SRR18191539, SRR18191528	Salting out	2206	O79:H48	1a, 2a	−	Unicycler	14,002	16	5,258,581	1,387,201	50.73	184	JAOQMH000000000
						Flye		6	5,257,865	5,088,236	50.71	184	JAOWPF000000000
SRR18191517, SRR18191506	Salting out	06QMA126.1	O153/O178:H19	1a, 2a, 2d	−	Unicycler	17,384	7	5,368,292	4,589,384	50.59	179	JAOQMG000000000
						Flye^*d*^		4	5,348,285	5,086,995	50.67	179	JAOWPE000000000
SRR18191495, SRR18191580	Salting out	06QMA140.2	O149:H1	1d	−	Unicycler	7,397	10	5,300,421	4,864,012	50.66	209	JAOQMF000000000
SRR18191569, SRR18191644	Bead based, salting out	06QMA181.1a	O91:H14	1a, 2b, 2b	−	Unicycler	28,527	13	5,618,742	5,174,383	50.74	242	JAOQME000000000
						Raven^*d*^		3	5,602,836	5,378,985	50.76	242	JAOWNJ000000000
SRR18191633, SRR18191566	Salting out	06QMA227.3	O116:H48	2a	−	Unicycler	13,538	5	4,987,186	4,797,877	50.62	230	JAOQMD000000000
SRR18191555, SRR18191544	Salting out	07HMPA386	O46/O134:H38	1a, 2a	−	Unicycler	18,809	5	4,943,051	4,790,646	50.57	150	JAOQMC000000000
						Flye^*h*^		10	4,965,900	4,764,076	50.63	150	JAOWPD000000000
SRR18191613, SRR18191610	Salting out	07HMPA903	O113:H21	2a, 2a	−	Unicycler	22,124	6	5,144,288	4,966,076	50.73	238	JAOQMB000000000
SRR18191609, SRR18191608	Salting out, bead based	07HMPA966	O91:H10	2a	−	Canu^*a*^	7,440	10	5,575,342	5,185,943	50.72	706	JAOWPP000000000
						Flye^*h*^		3	5,331,927	5,145,461	50.76	706	JAOWPC000000000
SRR18191607, SRR18191605	Salting out	09QMA299.3B	O175:H16	2a	−	Unicycler	25,581	7	5,088,106	4,812,392	50.73	207	JAOQMA000000000
						Raven^*h*^		4	5,087,513	4,862,173	50.73	207	JAOWNI000000000
SRR18191604, SRR18191603	Salting out	09QMA303.1	O8:H19	2g	−	Unicycler	20,236	3	5,132,730	5,022,134	50.76	168	JAOQLZ000000000
SRR18191602, SRR18191601	Salting out	09QMA311.2.3	O175:H16	2d	−	Unicycler	23,479	4	5,179,346	5,125,959	50.77	149	JAOQLY000000000
SRR18191600, SRR18191599	Salting out	09QMA33.1	O2:H27	2a	−	Unicycler	20,366	11	5,535,100	5,226,378	50.61	223	JAOQLX000000000
						Flye^*d*^		2	5,382,235	5,226,869	50.66	223	JAOWPB000000000
SRR18191542, SRR18191541	Salting out	09QMA47.1	O6:H10	1c	−	Unicycler	17,841	7	5,283,406	4,794,423	50.53	215	JAOQLW000000000
						Flye^*d*^		4	5,268,782	4,794,913	50.54	215	JAOWPA000000000
SRR18191540, SRR18191538	Salting out	1890-4	O26:H11	1a	+	Unicycler	22,209	17	5,801,088	4,709,082	50.64	272	JAOQLV000000000
						Raven^*d*^		2	5,834,018	5,743,918	50.68	272	JAOWNH000000000
SRR18191537, SRR18191536	Salting out	2011-O26	O26:H11	1a	+	Unicycler	17,173	33	5,891,761	2,948,002	50.45	262	JAOQLU000000000
						Flye^*e*^		9	5,906,758	5,674,537	50.51	262	JAOWOZ000000000
SRR18191535, SRR18191534	Salting out	2026-O145	O145:H28	2c	+	Unicycler	23,580	11	5,509,245	4,447,030	50.60	295	JAOQLT000000000
						Canu^*a*^		5	5,826,806	5,487,952	50.58	295	JAOWPO000000000
						Raven^*d*^		3	5,582,748	5,434,794	50.60	295	JAOWNG000000000
SRR18191533, SRR18191532	Salting out	2039-O26	O26:H11	1a	+	Raven	9,087	3	5,815,566	5,670,366	50.63	151	JAOWNF000000000
						Unicycler		21	5,735,378	3,340,315	50.52	151	JAOQLS000000000
SRR18191531, SRR18191530	Salting out	2048-O145	O145:H28	2c	+	Unicycler	18,920	15	5,526,494	5,203,884	50.57	250	JAOQLR000000000
						Flye^*d*^		10	5,628,166	5,402,071	50.59	250	JAOWOY000000000
SRR18191529, SRR18191527	Salting out	2142-O103	O103:H25	-^*m*^	+	Raven	22,773	10	5,661,007	5,368,150	50.51	174	JAOWNE000000000
SRR18191526, SRR18191525	Salting out	2149-O103	O103:H11	1a	+	Unicycler	14,099	27	5,844,869	5,519,265	50.69	153	JAOQLQ000000000
						Unicycler		12	5,758,941	5,122,111	50.69	153	JAOWNX000000000
SRR18191524, SRR18191523	Salting out	2236-3b	O26:H11	1a	+	Flye^*e*^	16,957	23	5,997,576	5,003,748	50.63	154	JAOWOX000000000
						Flye		30	6,044,444	5,011,133	50.61	154	JAOWNW000000000
SRR18191522, SRR18191521	Bead based	2918-O145	O145:H28	2a	+	Raven^*h*^	6,716	7	5,553,909	3,535,429	50.63	72	JAOWND000000000
						Unicycler		28	5,492,229	1,347,297	50.54	72	JAOQLP000000000
SRR18191520, SRR18191519	Salting out	3273-O103	O103:H2	1a	+	Flye^*g*^	20,998	22	5,769,060	5,373,151	50.72	238	JAOWOW000000000
SRR21721513, SRR18191516	Bead based, salting out	3313-O111	O111:H8	1a	+	Flye^*h*^	4,229	7	5,570,495	5,272,882	50.63	90	JAOWOV000000000
SRR21721512, SRR18191516	Bead based, salting out	3313-O111	O111:H8	1a	+	Unicycler	10,277	17	5,489,297	3,617,166	50.55	90	JAOQLO000000000
SRR18191518, SRR18191516	Salting out	3313-O111	O111:H8	1a	+	Unicycler	21,079	19	5,488,508	1,232,028	50.55	398	JAOWNV000000000
SRR18191515, SRR18191514	Salting out	3382-O103	O103:H2	1a	+	Flye^*f*^	13,920	18	5,719,857	4,395,063	50.65	128	JAOWOU000000000
						Unicycler		3	5,540,746	5,463,884	50.67	128	JAOWNL000000000
SRR18191513, SRR18191512	Bead based	3383-O103	O103:H2	1a	+	Flye^*b*^	5,882	18	5,719,857	4,395,063	50.65	671	JAOWNU000000000
						Flye^*e*^		5	5,542,730	5,454,423	50.65	671	JAOWOT000000000
SRR18191511, SRR18191510	Bead based	3383-O26b	O26:H11	1a	+	Flye	6,945	6	5,838,831	5,709,619	50.59	778	JAOWNT000000000
						Flye^*e*^		9	5,822,451	5,697,780	50.62	778	JAOWOS000000000
SRR18191509, SRR18191508	Bead based	3430-O103	O103:H2	1a	+	Flye^*e*^	8,683	5	5,557,311	4,743,383	50.73	259	JAOWOR000000000
						Flye^*b*^		6	5,558,675	4,744,103	50.72	259	JAOWNS000000000
SRR18191507, SRR21721511	Bead based, salting out	429-O26	O26:H11	1a	+	Unicycler	12,758 12,758	37	5,728,320	1,975,710	50.56	270	JAOQLN000000000
SRR18191507, SRR18191505	Salting out	429-O26	O26:H11	1a	+	Flye^*i*^		12	5,805,978	3,820,369	50.73	148	JAOWOQ000000000
						Unicycler		28	5,659,566	1,082,226	50.60	148	JAOWNR000000000
SRR18191504, SRR18191503	Salting out	4712-O26	O26:H11	1a	+	Flye^*h*^	25,662	4	5,972,468	5,737,945	50.75	71	JAOWOP000000000
SRR18191502, SRR18191501	Salting out	4747-O26	O26:H11	1a	+	Unicycler	12,482	39	5,664,587	2,049,284	50.56	148	JAOQLM000000000
						Canu^*a*^		6	5,856,627	5,621,430	50.64	148	JAOWPN000000000
						Flye^*i*^		6	5,714,135	5,609,626	50.62	148	JAOWOO000000000
SRR18191500, SRR18191499	Salting out	6423-O26	O26:H11	1a	+	Canu^*a*^	19,317	5	5,992,428	5,821,752	50.66	264	JAOWPM000000000
SRR18191498, SRR18191497	Bead based	97HMPL449	O3:H12	1a	−	Unicycler	5,941	6	5,371,299	5,104,752	50.51	334	JAOQLL000000000
						Canu^*a*^		7	5,472,012	3,671,848	50.47	334	JAOWPL000000000
SRR18191496, SRR18191590	Salting out	97HMPL473	O3:H12	1a	−	Unicycler	15,334	6	5,309,043	5,043,017	50.54	185	JAOQLK000000000
SRR18191589, SRR18191588	Bead based, salting out	97HMPL650	O110:H9	1c	−	Unicycler	28,759	4	4,862,109	4,701,590	50.71	141	JAOQLJ000000000
						Raven^*h*^		1	4,849,707	4,849,707	50.71	141	JAOWNC000000000
SRR18191587, SRR18191586	Bead based, salting out	97HMPL652	O110:H9	1c	−	Unicycler	22,595	3	4,864,116	4,850,996	50.71	238	JAOQLI000000000
SRR18191585, SRR21721510	Bead based, salting out	97HMPL657	O136:H12	1a	−	Unicycler	21,241	9	5,439,851	5,167,313	50.67	175	JAOWNQ000000000
SRR18191585, SRR18191584	Salting out	97HMPL657	O136:H12	1a	−	Flye^*d*^	21,241	44	5,655,428	5,156,955	50.68	125	JAOWON000000000
SRR18191583, SRR18191582	Bead based	97HMPL915	O112ac:H19	2c	−	Unicycler	8,149	2	5,247,946	5,142,912	50.78	805	JAOQLH000000000
SRR18191581, SRR18191579	Bead based	98HMPL324	O174:H21	2c	−	Raven^*d*^	5,263	4	5,214,333	5,042,330	50.76	908	JAOWNB000000000
						Canu		8	5,317,997	5,045,734	50.79	908	JAOWPK000000000
SRR18191578, SRR18191577	Salting out	98HMPL325	O17/O44/O77:H18	2d	−	Unicycler	14,916	6	5,373,540	5,090,743	50.71	199	JAOQLG000000000
SRR18191576, SRR18191575	Bead based	98HMPL475	O110:H9	1c	−	Unicycler	4,854	6	5,010,608	4,847,085	50.59	1148	JAOQLF000000000
SRR18191574, SRR18191573	Salting out	98HMPL479	O91:H14	1a, 2b, 2b	−	Unicycler	8,894	13	5,831,137	5,558,854	50.68	152	JAOQLE000000000
						Canu^*a*^		11	6,118,277	5,606,901	50.72	152	JAOWPJ000000000
SRR18191572, SRR18191571	Salting out	98HMPL487	O136:H12	1a	−	Unicycler	20,281	2	5,430,616	5,174,002	50.58	191	JAOQLD000000000
SRR18191570, SRR18191568	Salting out	Crl154393	O157:H7	2a, 2c	+	Unicycler	21,282	11	5,744,891	5,126,924	50.47	134	JAOQLC000000000
						Flye^*h*^		3	5,807,355	5,666,998	50.47	134	JAOWOM000000000
						Raven^*d*^		3	5,780,274	5,273,188	50.49	134	JAOWNA000000000
SRR18191567, SRR18191598	Salting out	Crl154395	O157:H7	2c	+	Unicycler	21,865	17	5,720,557	4,991,949	50.39	129	JAOQLB000000000
						Flye^*d*^		2	5,644,625	5,555,517	50.48	129	JAOWOL000000000
SRR18191597, SRR18191596	Salting out	Crl154397	O157:H7	2c, 2c	+	Unicycler	20,389	19	5,749,327	5,423,006	50.49	240	JAOQLA000000000
SRR18191595, SRR18191594	Salting out	Crl169922	O82:H8		−	Unicycler	20,244	6	4,868,876	2,456,854	50.78	225	JAOQKZ000000000
						Flye^*d*^		9	4,976,199	4,761,119	50.80	225	JAOWOK000000000
SRR18191593, SRR18191592	Salting out	Crl169937	O51:H41		−	Unicycler	13,654	4	4,958,903	4,892,016	50.63	95	JAOQKY000000000
SRR18191591, SRR18191643	Salting out	CrlO103	O103:H2		−	Unicycler	16,776	4	5,090,231	4,969,612	50.57	144	JAOQKX000000000
SRR18191642, SRR18191641	Salting out	CrlO157	O157:H7	2c	+	Unicycler	22,759	19	5,624,392	4,232,969	50.43	278	JAOQKW000000000
						Flye^*d*^		10	5,642,849	5,231,425	50.52	278	JAOWOJ000000000
SRR18191640, SRR18191639	Bead based, salting out	E. coli 12-1	O157:H7	2a, 2c	+	Canu^*a*^	25,609	3	5,909,043	5,747,499	50.44	284	JAOWPI000000000
						Flye^*e*^		4	5,807,203	5,689,286	50.50	284	JAOWOI000000000
SRR18191638, SRR18191637	Salting out	EC14	O174:H21	2c	−	Unicycler	20,105	4	5,082,783	4,945,157	50.83	219	JAOQKV000000000
						Flye^*h*^		12	5,155,830	4,911,711	50.83	219	JAOWOH000000000
SRR18191636, SRR18191635	Salting out, bead based	ECA135	O22:H16	2b, 2c, 2d	−	Canu^*a*^	29,351	5	5,459,604	5,386,935	50.63	276	JAOWPH000000000
						Flye^*h*^		1	5,313,712	5,313,712	50.70	276	JAOWOG000000000
SRR18191634, SRR18191632	Bead based, salting out	ECA15	O79:H48	1a, 2a	−	Raven^*h*^	19,793	5	5,326,335	4,303,608	50.71	58	JAOWMZ000000000
SRR18191631, SRR18191630	Salting out	ECA193	O8:H21	1a, 2c	−	Unicycler	20,554	9	5,596,707	5,158,603	50.69	222	JAOQKU000000000
SRR18191629, SRR18191628	Bead based	ECA194	O179:H8	1a, 2a	−	Unicycler	15,826	3	5,094,287	4,913,760	50.72	154	JAOQKT000000000
SRR18191627, SRR18191626	Bead based	ECA279	O174:H2	1a, 2d	−	Unicycler	2,565	7	5,126,030	2,145,451	50.72	199	JAOQKS000000000
						Flye^*a*^		4	5,167,400	5,037,956	50.76	199	JAOWOF000000000
SRR18191625, SRR18191624	Bead based, salting out	ECA329	O91:H21	2a, 2d	−	Unicycler	15,119	5	5,167,518	5,016,862	50.71	235	JAOWNP000000000
SRR18191625, SRR21748932	Salting out	ECA329	O91:H21	2a, 2d	−	Flye^*c*^	15,119	25	5,342,559	4,901,208	50.76	167	JAOWOE000000000
SRR18191565, SRR18191564	Salting out	ECA34	O5:H9	1a	+	Flye^*i*^	6,208	14	5,341,159	4,796,583	50.64	86	JAOWOD000000000
SRR18191623, SRR18191564	Salting out	ECA34	O5:H9	1a	+	Raven^*d*^	24,947	4	5,414,456	4,637,316	50.68	115	JAOWMY000000000
SRR18191563, SRR18191562	Bead based, salting out	ECA36	O5:H9	1a, 1a	+	Unicycler	22,228	14	5,418,653	4,566,021	50.66	100	JAOWNN000000000
SRR18191561, SRR18191560	Bead based, salting out	ECA37	O5:H9	1a, 1a	+	Flye^*h*^	24,413	11	5,403,523	5,306,942	50.69	112	JAOWOC000000000
SRR18191559, SRR18191558	Bead based	ECA399	O174:H21	2c	−	Unicycler	3,390	5	5,141,946	3,448,145	50.78	479	JAOQKR000000000
SRR18191557, SRR18191556	Salting out	ECA400	O6:H10	2d	−	Canu^*a*^	24,727	2	5,061,078	5,053,258	50.68	200	JAOWPG000000000
SRR18191554, SRR18191553	Bead based	ECA447	O22:H16	1a, 2b, 2d	−	Unicycler	6,419	5	5,351,044	5,298,558	50.63	121	JAOQKQ000000000
						Raven^*d*^		2	5,345,303	3,529,088	50.64	121	JAOWMX000000000
SRR18191552, SRR18191551	Salting out	ECA89	OgN-RKI2:H16	1c	−	Flye	18,224	52	5,336,292	4,922,938	50.72	180	JAOWNY000000000
						Unicycler		3	5,163,781	4,943,802	50.76	180	JAOWNK000000000
SRR18191550, SRR18191549	Salting out	ECA97	O113:H4	2d	−	Unicycler	17,597	4	5,262,159	5,257,847	50.55	149	JAOQKP000000000
SRR18191548, SRR18191547	Salting out	HSVR03	O157:H7	2c	+	Raven^*h*^	22,885	40	5,585,011	1,473,641	50.49	67	JAOWMW000000000
SRR18191546, SRR18191545	Salting out	HSVR04	O157:H7	2c	+	Flye^*h*^	22,926	11	5,843,094	5,515,994	50.43	113	JAOWOB000000000
SRR18191543, SRR18191622	Bead based	NC672mucus	Ox13:H2		−	Flye^*a*^	6,632	3	5,323,626	5,187,741	50.66	381	JAOWOA000000000
SRR18191621, SRR18191620	Salting out	NC809	O41:H7		−	Unicycler	19,581	8	5,227,833	4,891,608	50,51	230	JAOQKO000000000
SRR18191619, SRR18191618	Bead based	NV34	O168:H8	2d	−	Unicycler	6,450	6	5,269,996	4,249,978	50,77	402	JAOQKN000000000
						Raven^*d*^		3	5,314,568	5,184,508	50.81	402	JAOWMV000000000
SRR18191617, SRR18191616	Bead based	NV36	O75:H8	1c,2b	−	Unicycler	6,484	8	5,575,909	5,306,095	50.65	468	JAOQKM000000000
						Unicycler^*l*^		11	5,577,269	5,301,141	50.65	113	JAOWNM000000000
SRR18191615, SRR18191614	Salting out	Slk8430761	O177:H25	2c, 2d	+	Flye^*b*^	20,113	28	5,639,340	5,231,455	50.58	180	JAOWNO000000000
						Flye^*h*^		30	5,658,335	5,248,496	50.58	180	JAOWNZ000000000
SRR18191612, SRR18191611	Salting out	Slk8430767	O177:H25	2c, 2d	+	Unicycler	17,583	12	5,420,630	5,017,089	50.54	150	JAOQKL000000000
						Raven^*d*^		3	5,458,939	5,276,849	50.57	150	JAOWMU000000000

a Long-read assembly polished with pilon.

b Long-read assembly polished with racon and medaka.

c Long-read assembly polished with racon (2 rounds) and medaka.

d Long-read assembly polished with racon, medaka, and pilon.

e Long-read assembly polished with racon, medaka, and pilon, and contigs <1 kb were removed.

f Long-read assembly polished with racon (3 rounds), medaka, and pilon, and contigs <1 kb were removed.

g Long-read assembly polished with racon (4 rounds), medaka, and pilon, and contigs <1 kb were removed.

h Long-read assembly polished with racon (3 rounds) and pilon.

i Long-read assembly polished with racon (3 rounds), pilon, and contigs <1 kb were removed.

j If two methods were used for DNA extraction, the first one corresponds to the DNA extraction method used for sequencing using Illumina and the second for MinION sequencing.

k Serotypes were determined using *in silico* analysis.

l 50× subsampling.

m - are used to describe bash arguments/options.

Assemblies from 1 to 52 contigs (mean, 10.33 ± 9.73) with a total assembly length between 4,849,707 and 6,118,277 bp (mean, 5,468,530.42 ± 292,599.77 bp) and mean GC content of 50.64% (±0.13) were generated. *N*_50_ values ranged from 1,082,226 to 5,821,752 bp (mean, 4,759,454.32 ± 1,018,105.98 bp). The main sample characteristics, including serotype, *stx* and *eae* gene presence (determined using abricate [--min-cov 60 --min-id 80]), genome coverage, and accession numbers are reported in Table 1. All genomes were annotated using the National Center for Biotechnology Information (NCBI) Prokaryotic Genome Annotation Pipeline (PGAP) v6.3 at http://www.ncbi.nlm.nih.gov/genome/annotation_prok (12).

### Data availability.

Short and long raw reads were deposited in the NCBI SRA database under BioProject accession number PRJNA808207. Additionally, assembled genomes were deposited in GenBank under accession numbers as referred to in Table 1 within BioProject accession numbers PRJNA808207, PRJNA885284, PRJNA884285, PRJNA884276, and PRJNA883638.
